# Association between Platelet Counts before and during Pharmacological Therapy for Patent Ductus Arteriosus and Treatment Failure in Preterm Infants

**DOI:** 10.3389/fped.2018.00041

**Published:** 2018-03-07

**Authors:** Hannes Sallmon, Sven C. Weber, Juliane Dirks, Tamara Schiffer, Tamara Klippstein, Anja Stein, Ursula Felderhoff-Müser, Boris Metze, Georg Hansmann, Christoph Bührer, Malte Cremer, Petra Koehne

**Affiliations:** ^1^Department of Neonatology, Charité University Medical Center, Berlin, Germany; ^2^Department of Pediatrics, Neonatology and Pediatric Intensive Care Medicine, University of Greifswald, Greifswald, Germany; ^3^Division of Neonatology, Department of Pediatrics I, University Hospital Essen, Essen, Germany; ^4^Department of Pediatric Cardiology and Critical Care, Hannover Medical School, Hannover, Germany

**Keywords:** preterm infant, ibuprofen, indomethacin, patent ductus arteriosus, platelets, ductal closure

## Abstract

**Background:**

The role of platelets for mediating closure of the ductus arteriosus in human preterm infants is controversial. Especially, the effect of low platelet counts on pharmacological treatment failure is still unclear.

**Methods:**

In this retrospective study of 471 preterm infants [<1,500 g birth weight (BW)], who were treated for a patent ductus arteriosus (PDA) with indomethacin or ibuprofen, we investigated whether platelet counts before or during pharmacological treatment had an impact on the successful closure of a hemodynamically significant PDA. The effects of other factors, such as sepsis, preeclampsia, gestational age, BW, and gender, were also evaluated.

**Results:**

Platelet counts before initiation of pharmacological PDA treatment did not differ between infants with later treatment success or failure. However, we found significant associations between low platelet counts during pharmacological PDA therapy and treatment failure (*p* < 0.05). Receiver operating characteristic (ROC) curve analysis showed that platelet counts after the first, and before and after the second cyclooxygenase inhibitor (COXI) cycle were significantly associated with treatment failure (area under the curve of >0.6). However, ROC curve analysis did not reveal a specific platelet cutoff-value that could predict PDA treatment failure. Multivariate logistic regression analysis showed that lower platelet counts, a lower BW, and preeclampsia were independently associated with COXI treatment failure.

**Conclusion:**

We provide further evidence for an association between low platelet counts during pharmacological therapy for symptomatic PDA and treatment failure, while platelet counts before initiation of therapy did not affect treatment outcome.

## Introduction

Ductus arteriosus (DA) closure is an important step during the transition from fetal to extrauterine neonatal life. Failure of spontaneous DA closure in immature infants can be associated with several complications such as left ventricular volume overload, pulmonary edema, and impairment of lung compliance (1). However, due to a high spontaneous closure rate and a favorable outcome of infants who were discharged home with a persistently patent DA, it is controversial which patent ductus arteriosus (PDAs) require treatment (2). The aforementioned associations between adverse outcomes and a large and significant PDA provide the rationale for many clinicians to initiate treatment for hemodynamically significant PDAs (hsPDA) (3). The current mainstay of therapy is the administration of the cyclooxygenase (COX) inhibitors ibuprofen or indomethacin. These drugs act by inhibiting COX involved in producing prostaglandins E1 and E2 that are known to promote ductal patency. The success rate of this pharmacological approach, however, varies and is difficult to predict for individual infants (1, 3).

In mice, platelet-triggered ductal sealing and subsequent vascular remodeling appears to be an important mechanism for definite DA closure (4). Murine platelets are recruited to the endothelium of the DA soon after birth, and platelet dysfunction or defective platelet formation was found to result in persistently patent DA. The role of platelets for DA closure in humans, however, remains controversial, especially in preterm infants. An association between low platelet counts on the first day of life and PDA has been described in several retrospective analyses (4–7). However, similar analyses failed to confirm these findings (8–13). Two recent meta-analyses indicated weak, but significant, associations between hsPDA and thrombocytopenia on the first day of life and pre-treatment thrombocytopenia and pharmacological treatment failure, respectively (14, 15).

However, data from these studies on platelet counts during and just before initiation of therapy by day of life 4 or later are sparse in the reported investigations (15, 16). We herein analyzed whether platelet counts before initiation and during pharmacological PDA therapy are associated with treatment failure in a large cohort of very low birth weight infant (VLBW) infants.

## Materials and Methods

### Patients

This retrospective cohort study was conducted at the neonatal intensive care units (NICUs) in the Department of Neonatology, Campus Virchow-Klinikum and Campus Mitte, Charité University Medical Center, Berlin Germany (1998–2008). All VLBW infants born in the respective period were included if they had received pharmacological treatment for hsPDA. Platelet counts before initiation of therapy, after the first, and before and after a second or third treatment cycle (if administered) were recorded, and the incidence of treatment success in the different groups was calculated. The clinical risk index for babies (CRIB) was calculated and parental consent for data collection was obtained after patient admission to the NICUs.

### PDA Diagnosis/Algorithm for PDA Intervention

Infants were examined for hsPDA on days of (postnatal) life (DOL) 4–5 and when clinically indicated. Echocardiographic evaluation included shunt direction of a PDA in color Doppler mode (high upper parasternal short axis) as well as the minimal internal ductal diameter of three to five measurements taken in B-mode. The left atrium to aortic root (LA/Ao) ratio was measured by M-mode (parasternal long axis). The Doppler measurement of the resistance index in the anterior cerebral artery was done at the same time. Examinations were performed as previously described (8).

Cyclooxygenase inhibitor (COXI) treatment was initiated in all VLBW infants with an hsPDA. A PDA with left-to-right shunt was considered hemodynamically significant if (i) a respiratory set back with a supplemental oxygen requirement >30% and/or mechanical ventilation, (ii) a LA/Ao ratio ≥1.4 in the echocardiogram and/or (iii) ductal diameter ≥2.5 mm, and/or (iv) a decreased end-diastolic flow in the anterior cerebral artery with a resistance index ≥0.85 in the cerebral ultrasound were present. There has been no substantial change in the clinical and echocardiographic criteria used to determine the need for PDA intervention during the study period.

### COXI Therapy

None of the infants received treatment before an echocardiogram was performed. Infants received indomethacin or ibuprofen (Pedea^®^) exactly as previously described in detail (including dosing, duration, etc.) (8). Successful response to COXI treatment was defined as absent ductal shunt flow 24–48 h after therapy, all other cases were defined as COXI treatment failure. Echocardiographic reassessment was performed after each therapy cycle.

### Statistics

Normality was tested by Kolmogorov–Smirnov test. Comparisons between groups were made by Kruskal–Wallis or Mann–Whitney *U*-test as appropriate for continuously scaled data and by chi-square test for categorical data. *p* Values <0.05 were considered statistically significant. Statistical analyses, including multivariate logistic regression and receiver operating characteristic (ROC) curve analyses, were carried out using the IBM SPSS Statistics 19 software.

## Results

### Incidence of PDA and Treatment Success Rates

**Figure 1:**
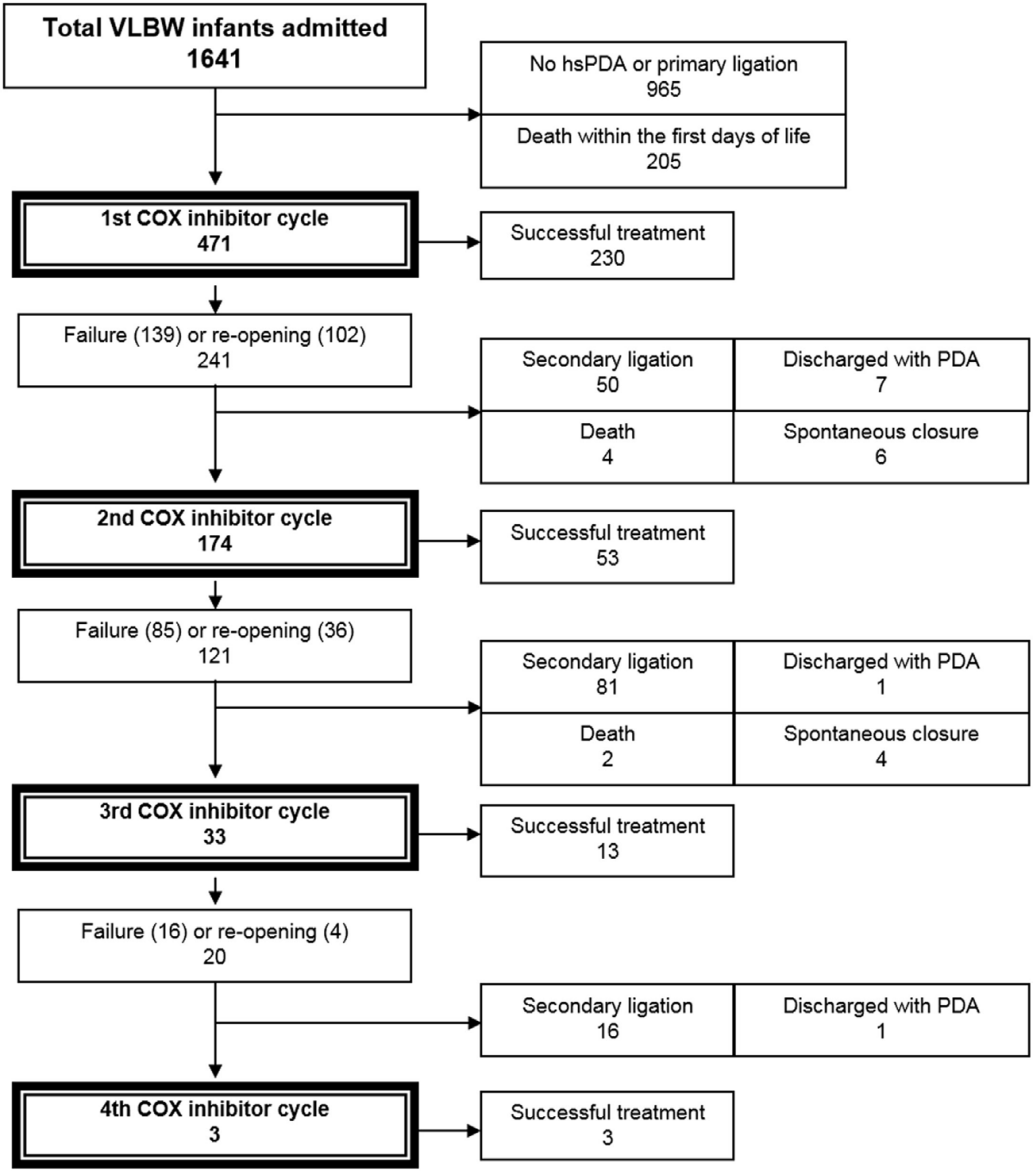
Flowchart: study population. Treatment with COX inhibitors was considered successful, if no further PDA therapy was required. COX, cyclooxygenase; hsPDA, hemodynamically significant PDA; PDA, patent ductus arteriosus; VLBW, very low birth weight (<1,500 g).

Out of a total of 1,641 VLBW infants, we included 471 patients (28.7%) who were treated with COXI for hsPDA in the respective period (including 353 extremely low birth weight infant infants, Figure 1). Demographics of the patient cohort are shown in Table 1. The first COXI course was permanently successful in 48.8% (230/471) of all VLBW infants. Of note, although 332 infants (70.5%) achieved initial DA closure after the first COXI course, 102 infants developed reopening of their PDA after the first treatment course. Out of the remaining 241 infants, 174 received a second, 33 a third, and 3 a fourth COXI course (Figure 1). In total, 299/471 infants (63.5%) achieved permanent ductal closure by pharmacological treatment, while 147 (31.2%) infants underwent secondary ligation. Nine patients (1.9%) were discharged home with a hsPDA, 6 (1.3%) died, and 10 (2.1%) experienced spontaneous PDA closure before a subsequent treatment cycle was initiated. Of note, treatment success rates between the infants that initially received indomethacin or ibuprofen were not significantly different (*p* > 0.05).

**Table 1:** Characteristics of VLBW infants undergoing pharmacological treatment by cyclooxygenase inhibitors (COXI) for hemodynamically significant patent ductus arteriosus.

VLBW infants	COXI Total	COXI Success	COXI Failure
*N* (%)	471 (100)	299 (63.5)	172 (36.5)
Female (*n*, %)	208 (44)	123 (41.1)	85 (49.4)
Birth weight (BW) (g)	867 (270–1.490)	875 (442–1.490)	749 (270–1.480)**
Gestational age (weeks)	26 (23–34)	26 (23–34)	25 (23–34)**
Twins (*n*, %)	125 (26.5)	78 (26.1)	47 (27.3)
Triplets (*n*, %)	25 (5.3)	16 (5.4)	9 (5.2)
Quadruplets (*n*, %)	3 (0.6)	1 (0.3)	2 (1.2)
Apgar 5 min	7 (1–9)	7 (1–9)	7 (1–9)
CRIB score	6 (0–17)	5 (0–17)	7 (0–17)**
Preeclampsia (*n*, %)	60 (12.7)	45 (15.1)	15 (8.7)*
Sepsis (*n*, %)	417 (88.5)	253 (84.6)	164 (95.3)*

Data are numbers (percentages) or median (range). In the top row, data in parentheses represent percentages referring to the total number of 471 included VLBW preterm infants (horizontal comparison within the first row). For all other data shown, percentages in parentheses refer to the total number in the upper first row of the same column (vertical comparison within the same column). Statistically significant values are given in bold (*p < 0.05; **p < 0.001).

*VLBW, very low birth weight infant (<1,500 g); COXI, cyclooxygenase inhibitor (indomethacin or ibuprofen); CRIB, clinical risk index for babies*.

### Platelet Counts before Initiation and during Therapy and Treatment Success Rates

The platelet counts before initial pharmacological treatment did not differ significantly between infants with and infants without successful treatment. However, the first platelet counts after completion of the first COXI course differed significantly between infants with initial treatment success and failure (*p* < 0.001). Similar results were obtained for the platelet counts just before (*p* < 0.05) and after the second COXI cycle (*p* < 0.05, Table 2). Trends toward higher platelet counts in the treatment success group were also seen before and after the third course of COXI treatment. However, these results did not reach statistical significance due to a lower *n* number.

**Table 2:** Platelet numbers in very low birth weight infants treated for hemodynamically significant PDA before and after each course of cyclooxygenase inhibitors.

	COXI success	COXI failure	Time point (day of life)
**Platelet counts before 1st COXI cycle**			
Median	201,000	196,000	3
Minimum	27,000	11,000	1
Maximum	608,000	531,000	52

**Platelet counts after 1st COXI cycle**			
Median	308,000	220,000**	11
Minimum	19,000	35,000	3
Maximum	812,000	633,000	60

**Platelet counts before 2nd COXI cycle**			
Median	309,000	233,000*	14
Minimum	56,000	12,000	4
Maximum	580,000	497,000	63

**Platelet counts after 2nd COXI cycle**			
Median	289,000	198,000*	19
Minimum	31,000	24,000	7
Maximum	598,000	634,000	52

**Platelet counts before 3rd COXI cycle**			
Median	354,000	200,000	20
Minimum	96,000	13,000	10
Maximum	631,000	502,000	66

**Platelet counts after 3rd COXI cycle**			
Median	328,500	181,000	30
Minimum	105,000	30,000	14

Platelet counts are given in number/μL. Statistically significant differences between treatment success and failure are given in bold (*p < 0.05; **p < 0.001).

*COXI, cyclooxygenase inhibitor*.

### ROC Curve and Logistic Regression Analyses

**Figure 2:**
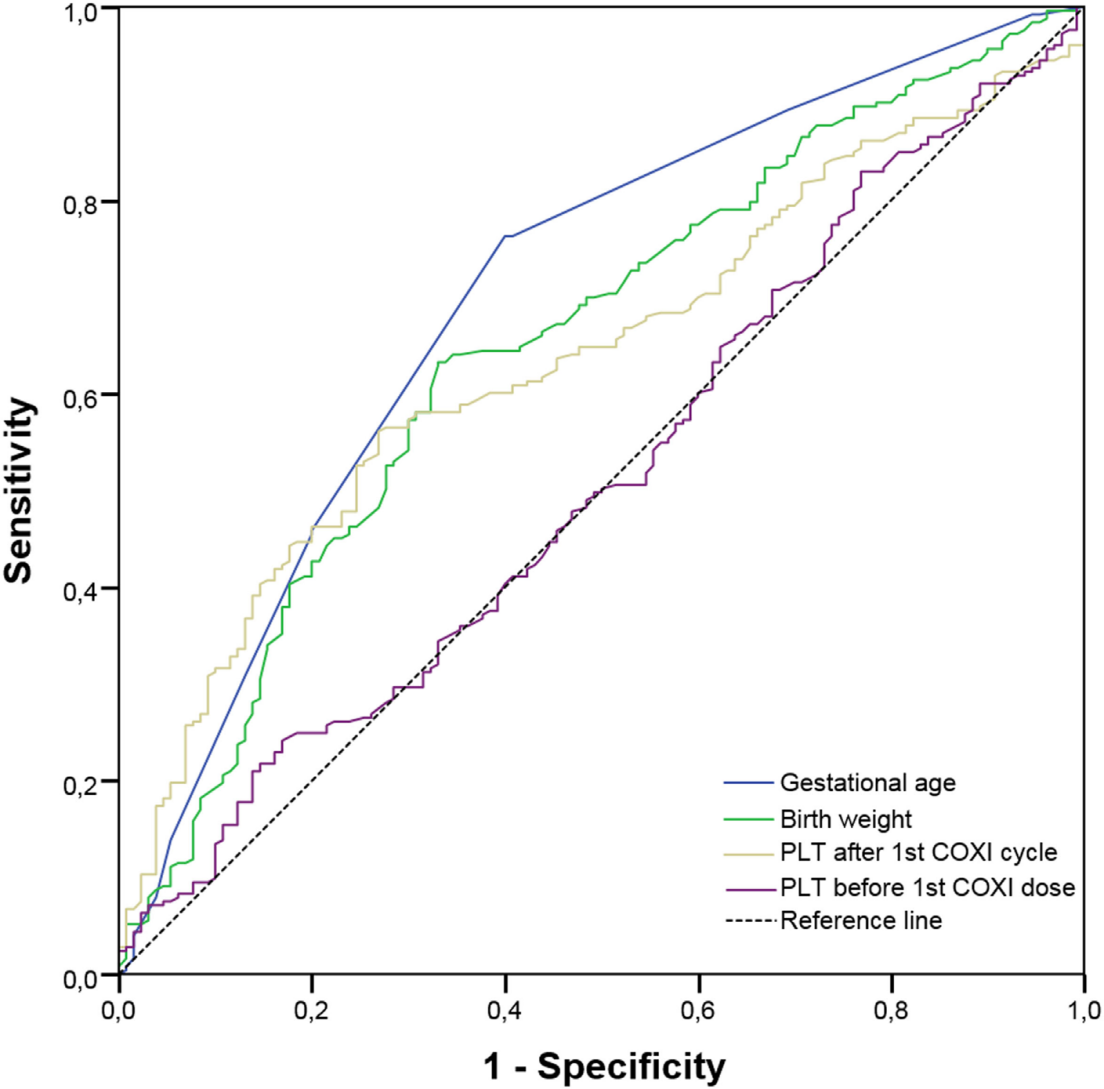
Receiver operating characteristic (ROC) curve analysis. The ROC curve analysis shows a significant association between platelet counts after the first cyclooxygenase inhibitor (COXI) cycle and treatment failure with an area under the curve (AUC) of 0.617 and a confidence interval (CI) of 0.560–0.674. However, the form of the curve indicated no specific cutoff that could be used to predict treatment failure below a specific platelet count. Of note, similar results were obtained for platelet counts before and after the second COXI cycle (data not shown). For comparison, we also included platelet counts before the first COXI dose (AUC 0.513; CI 0.453–0.574), gestational age (AUC 0.703; CI 0.647–0.759), and birth weight (AUC 0.652; CI 0.594–0.710).

Next, we performed a ROC curve analysis which showed a significant association between platelet counts after the first COXI cycle and treatment failure with an area under the curve (AUC) of 0.617 and a confidence interval (CI) of 0.560–0.674. However, the form of the curve indicated no specific cutoff that could be used to predict treatment failure below a specific platelet count (Figure 2). Of note, similar results were obtained for platelet counts before and after the second COXI cycle (data not shown).

In this study, preeclampsia was associated with significantly lower platelet counts at birth (*p* < 0.001). Similarly, infants with sepsis, defined by elevated systemic markers of inflammation (IL-6 and/or CRP) and the use of antibiotics, showed a non-significant trend toward lower platelet counts before initiation of therapy. Multivariate logistic regression analysis showed that higher platelet counts after the first COXI cycle were independently associated with treatment success, while sepsis, and gender had no significant effect (Table 3). In addition, birth weight (BW) [or gestational age (GA)], and preeclampsia were also independent predictors of treatment success rates.

**Table 3:** Logistic regression analysis for prediction of COXI treatment success.

	*B*	SE	Wald	df	Significance	Exp(B)
Gestational age***	0.294	0.073	16.140	1	**<0.001**	1.342
PLT after 1st COXI cycle***	0.003	0.001	12.629	1	**<0.001**	1.003
Preeclampsia*	−0.867	0.414	4.379	1	**0.036**	0.420
Sepsis/infection	0.315	0.464	0.460	1	0.497	1.370
Male gender	−0.306	0.237	1.669	1	0.196	0.736
Constant	−0.922	1.967	12.380	1	0.000	0.001

381/471 (80.9%) of cases with a complete dataset were included in this analysis. Of note, sepsis/infection was defined by elevated markers of infection and antibiotic therapy. Significant factors (*p < 0.05, ***p < 0.001) are listed in bold.

*B, coefficient for the constant; Wald, Wald chi-square test; df, degree of freedom; Exp(B), exponent B; PLT, platelet number; COXI, cyclooxygenase inhibitor*.

## Discussion

The role of platelets during closure of the DA is subject to ongoing debates. While experimental data suggest that platelets are involved in spontaneous ductal closure (4), the clinical implications of these findings are controversial especially in very immature infants. In our previous study on 1,350 VLBW infants, we found no association between platelet counts within the first 24 h after birth and spontaneous ductal closure. We also did not find an association between platelet count on DOL 1 and a later treatment failure. However, sepsis, male gender, a lower BW, or GA were risk factors for hsPDA development (8). Similarly, Shah et al. assessed serial platelet counts (DOL 1–7) in preterm infants born <28 weeks of gestation and PDA closure rates (*n* = 497). Neither high nor low platelet numbers had an impact on permanent DA closure. However, in this study, all infants received prophylactic indomethacin starting on DOL 1 (17). Data on the platelet counts during and just before initiation of symptomatic PDA therapy by day of life 4 or later are sparse in the reported investigations (15, 16).

In this study, we addressed this issue by investigating serial platelet counts in relation to PDA treatment success, which provides a differential picture on the influence of platelet counts before and after each COXI cycle and its success. We were able to demonstrate, that platelet counts just before initiation of pharmacological PDA therapy (median DOL 3) were not associated with treatment failure. However, we found statistically significant associations between platelet counts during COXI therapy and the respective success rates. Furthermore, a lower BW, a lower GA, and a higher CRIB score were significantly associated with treatment failure of COXI therapy in this cohort. Interestingly, male gender, which others and we previously reported to be an independent risk factor for development of an hsPDA, was not significantly associated with treatment failure rates. Similarly, preeclampsia, which is known to affect platelet numbers (18, 19), was significantly associated with COXI therapy failure. Interestingly, sepsis/infection had no effect on treatment success rates, which is likely due to the less strict definition used in this study (elevated systemic markers of infection, use of antibiotics).

The available data on platelet counts and their implications for DA closure and PDA treatment success are controversial. The reported heterogeneity of previous studies might be explained by different definitions of hsPDA, different echocardiographic screening protocols (parameters and time points), and by different treatment protocols (prophylactic vs. symptomatic treatment). This study cannot definitively answer the question of how clinically relevant low platelet counts are for ductal closure in human preterm infants. However, it provides evidence for an association between low platelet counts during COXI therapy initiated between DOL 3 and 5 for symptomatic PDA and treatment failure. Logistic regression analysis showed a significant albeit weak independent effect of platelet counts during PDA therapy on success rates. These findings currently do not change clinical practice as no clear cutoff value that was associated with treatment failure could be established.

Besides platelet numbers, thrombocyte function might play a role during DA closure. The most immature infants are also most likely to have impaired platelet function (e.g., due to sepsis, immaturity, or medication). Therefore, future prospective investigations should include parameters of platelet function (20), especially in infants receiving non-steroidal anti-inflammatory drugs (NSAIDs), which are known to potentially alter platelet function. In regard to this, Kahvecioglu et al. reported longer collagen-ADP times in infants with hsPDA (21). Other authors have suggested that parameters such as platelet mass or mean platelet volume might be important for DA closure (6, 11, 12, 21, 22).

NSAIDs are standard of care in closing a hsPDA in preterm infants (3). Overall, we observed successful ductal closure after initial therapy in 63.5% of all VLBW infants, respectively, which is consistent with the ranges reported in the literature (1). In the patients analyzed, NSAIDs were usually withheld if platelet counts were <50,000/μL. A total of 55 infants received platelet transfusions during COXI treatment, which did not influence the treatment success rates. NSAID treatment of infants with moderate thrombocytopenia (50,000–100,000/μL) was considered save. However, this assumption was based on the only randomized controlled trial on platelet transfusion thresholds performed to date. Andrew et al. randomized 152 thrombocytopenic infants to receive platelet transfusions for any platelet count <150,000/μL or for a platelet count <50,000/μL (or clinical indications) targeting a primary outcome of IVH. These investigators found no significant differences in frequency or severity of IVH between the two groups (23). However, Brunner et al. reported an increased risk for intracerebral bleeding in VLBW infants with moderate thrombocytopenia that were treated with COXI (24).

These findings illustrate the critical need for well-designed prospective randomized trials that investigate the outcome of thrombocytopenic neonates and the safety of different platelet transfusion thresholds (19, 25, 26). Furthermore, trials investigating the optimal PDA management (indication for treatment, optimal dosing of NSAIDs, etc.) are essential to improve outcomes, of the most immature infants.

## Ethics Statement

This study was carried out in accordance with the recommendations of the local ethics committee at Charité Berlin. Formal ethics approval for this specific study was not required due to the retrospective nature of the investigations.

## Author Contributions

HS, GH, and PK conceptualized the study. HS, SW, PK, TK, JD, and TS collected the data. HS, SW, BM, PK, JD, and GH analyzed the data. HS wrote the first draft of the manuscript. All authors contributed to the interpretation of the data and critically reviewed and contributed to the final draft of the manuscript.

## Conflict of Interest Statement

The authors declare that the research was conducted in the absence of any commercial or financial relationships that could be construed as a potential conflict of interest. The authors have no financial relationships relevant to this article to disclose.

## Abbreviations

BW, birth weight; CBC, complete blood count; COX, cyclooxygenase; COXI, cyclooxygenase inhibitor; CRIB, clinical risk indek for babies; DA, ductus arteriosus; DOL, days of (postnatal) life; ELBW, extremely low birth weight infant; GA, gestational age; PDA, patent ductus arteriosus; hsPDA, hemodynamically significant PDA; NICU, neonatal intensive care unit; NSAID, non-steroidal anti-inflammatory drug; ROC, receiver operating characteristic; VLBW, very low birth weight infant.
